# Underpowered samples, false negatives, and unconscious learning

**DOI:** 10.3758/s13423-015-0892-6

**Published:** 2015-06-30

**Authors:** Miguel A. Vadillo, Emmanouil Konstantinidis, David R. Shanks

**Affiliations:** Primary Care and Public Health Sciences, King’s College London, Capital House, 42 Weston St., London, SE1 3QD UK; Department of Social and Decision Sciences, Carnegie Mellon University, 5000 Forbes Avenue, Pittsburgh, PA BP 208 USA; Division of Psychology and Language Sciences, University College London, 26 Bedford Way, London, WC1H 0AH UK

**Keywords:** Contextual cuing, False negatives, Implicit learning, Null hypothesis Significance testing· Statistical power

## Abstract

The scientific community has witnessed growing concern about the high rate of false positives and unreliable results within the psychological literature, but the harmful impact of false negatives has been largely ignored. False negatives are particularly concerning in research areas where demonstrating the absence of an effect is crucial, such as studies of unconscious or implicit processing. Research on implicit processes seeks evidence of above-chance performance on some implicit behavioral measure at the same time as chance-level performance (that is, a null result) on an explicit measure of awareness. A systematic review of 73 studies of contextual cuing, a popular implicit learning paradigm, involving 181 statistical analyses of awareness tests, reveals how underpowered studies can lead to failure to reject a false null hypothesis. Among the studies that reported sufficient information, the meta-analytic effect size across awareness tests was *d*_*z*_ = 0.31 (95 % CI 0.24–0.37), showing that participants’ learning in these experiments was conscious. The unusually large number of positive results in this literature cannot be explained by selective publication. Instead, our analyses demonstrate that these tests are typically insensitive and underpowered to detect medium to small, but true, effects in awareness tests. These findings challenge a widespread and theoretically important claim about the extent of unconscious human cognition.

Research practices in the behavioral sciences are under scrutiny to an extent that would have been inconceivable 10 years ago. Much of the debate has concerned habits (such as “*p*-hacking” and the filedrawer effect) which can boost the prevalence of false positives in the published literature (Ioannidis, Munafò, Fusar-Poli, Nosek, & David, 2014; Simmons, Nelson, & Simonsohn, 2011). Much less attention has been paid to the harmful consequences of false negatives, namely reports which purport to present evidence supporting false null hypotheses (Fiedler, Kurtzner, & Krueger, 2012). Via meta-analysis of a particular sub-literature within the field of implicit learning, we demonstrate how the use of underpowered experiments and Null Hypothesis Significance Testing (NHST) can combine to encourage the reporting of false negatives and consequent theoretical distortion.

When a researcher obtains a result that is significant at *p* < .05 and consequently reports that the null hypothesis is rejected, then of course we have learned something: That the likelihood of obtaining data at least as extreme as those that were observed, if the null hypothesis is true, is less than 5 %. Many would argue that we have not learned very much – for example, we have not learned that the null hypothesis is false or unlikely (Dienes, 2011; Fidler & Loftus, 2009). In contrast, when the researcher finds a result that is not significant (*p* > .05) and consequently concludes that the null hypothesis cannot be rejected, from the point of view of NHST we have learned literally nothing. We have not learned that the experimental hypothesis is false (the experiment may be underpowered) nor have we learned that the null hypothesis is true. Thus there is a sense in which any conclusions drawn from failures to reject the null hypothesis are intrinsically more problematic than those drawn from rejections of the null.

Underpowered studies are a major contributing factor to the reporting of both false positives and false negatives (Button et al., 2013). The power of typical studies in psychology, combined with typical effect sizes, indicates that the literature contains far more significant results than it should, suggesting that it is therefore biased in favor of significant findings (false positives) rejecting true null hypotheses (Francis, 2012). But low power might also contribute to the reporting of false negatives, when authors wish to demonstrate the absence of some effect. For instance, the absence of judgmental biases outside the laboratory (e.g., List, 2002), the absence of gender differences in math performance (e.g., Hyde, Lindberg, Linn, Ellis, & Williams, 2008), the absence of differences between studies run in the laboratory versus online (McGraw, Tew, & Williams, 2000), the absence of awareness in studies of implicit processing, and many other such influential claims depend on null effects which could potentially be false negatives if based on low-powered studies. NHST provides further impetus, in that its dichotomous nature (significant/nonsignificant at the arbitrary *p* = .05 cliff-edge) and focus on rejection of the null hypothesis encourage both researchers and students to interpret failure to reject the null hypothesis as implying that the null hypothesis is true (Hoekstra, Finch, Kiers, & Johnson, 2006). As Fidler and Loftus (2009) note, “this kind of almost irresistible logical slippage can, and often does, lead to all manner of interpretational mischief later on” (p. 29).

Confidence intervals (CIs) have an important role to play in the interpretation of null results (but see Hoekstra, Morey, Rouder, & Wagenmakers, 2014). If such intervals include zero but are narrow, then it can safely be concluded that the effect in question is either small or negligible in magnitude (though of course it cannot be concluded that the effect is non-existent). But if the intervals are wide, then little confidence can be placed on the null result and a motivation is provided for running larger sample sizes. Equally important is the role that meta-analysis can play in reaching valid conclusions across bodies of research featuring null results. Even though individual underpowered studies may fail to reject the null hypothesis, meta-analysis across a set of such studies may permit modest but real effects to be detected.

In the present research we illustrate these issues via a systematic review of a large body of studies within the field of implicit learning. These studies depend crucially on null results in awareness checks, because implicit learning by definition involves mental processing in the absence of awareness. As we show, the majority of these studies are underpowered to detect small but real awareness effects. We illustrate how the computation of CIs (and their graphic depiction) and meta-analysis can lead to radically different conclusions from those reached in the individual studies themselves. Our results challenge a theoretically crucial conclusion drawn from this body of research.

## Null results as a crucial feature of research on implicit processing

Research on implicit processing provides an excellent example to illustrate the consequences of overreliance on NHST to gather support for the null hypothesis. In a typical experiment on implicit processing, participants’ performance on some task is above a baseline level, but this behavioral outcome is seemingly not accompanied by any awareness of the environmental cues or regularities that gave rise to the behavior. For instance, in research on subliminal perception, some form of behavior is primed by a briefly-flashed stimulus of which participants are unaware (e.g., Dehaene et al., 1998); research in neuropsychology suggests that perception, memory, and choices can be influenced by cues unconsciously in various patient populations (Bechara et al., 1995; Cohen & Squire, 1980; Goodale, Milner, Jakobson, & Carey, 1991); in research on behavior priming, some behavioral response such as voting intentions (Hassin, Ferguson, Shidlovski, & Gross, 2007), walking speed (Bargh, Chen, & Burrows, 1996), or answering general knowledge questions (Dijksterhuis & van Knippenberg, 1998) is influenced by a subtle cue without participants being aware of this influence; research on implicit moral judgments, emotions, and attitudes proposes that behaviors in each of these domains can again be influenced by environmental cues unconsciously (Bargh, 2006; Williams & Bargh, 2008), and so on. Usually the absence of awareness is inferred from a null result in an awareness test (Dienes, 2015). For example, participants might fail to detect stimuli in a forced-choice test or they might perform at chance when asked to exert some control over the cue’s influence on their behavior.

However, as mentioned above, null results in NHST are inherently ambiguous. They can mean either that the null hypothesis is true or that there is insufficient evidence to reject it. In the context of implicit processing experiments, this means that when an awareness test yields a non-significant result, this can indicate either that participants were really unconscious of the cue or that the awareness test is inadequate to permit a firm conclusion about whether participants were aware or not. Unfortunately, the statistical analyses reported in many implicit processing experiments are insufficient to test which of these two interpretations is more plausible. A Bayesian approach to statistical analysis might allow researchers to quantify to what extent null results reflect a real absence of effects or a lack of statistical sensitivity (Dienes, 2015; Rouder, Speckman, Sun, Morey, & Iverson, 2009). However, these Bayesian analyses are seldom conducted (or reported) on data from awareness tests. Furthermore, researchers sometimes report so little information in their statistical analyses that it is also difficult for other researchers to compute these Bayesian analyses on reported data.

**Fig. 1:**
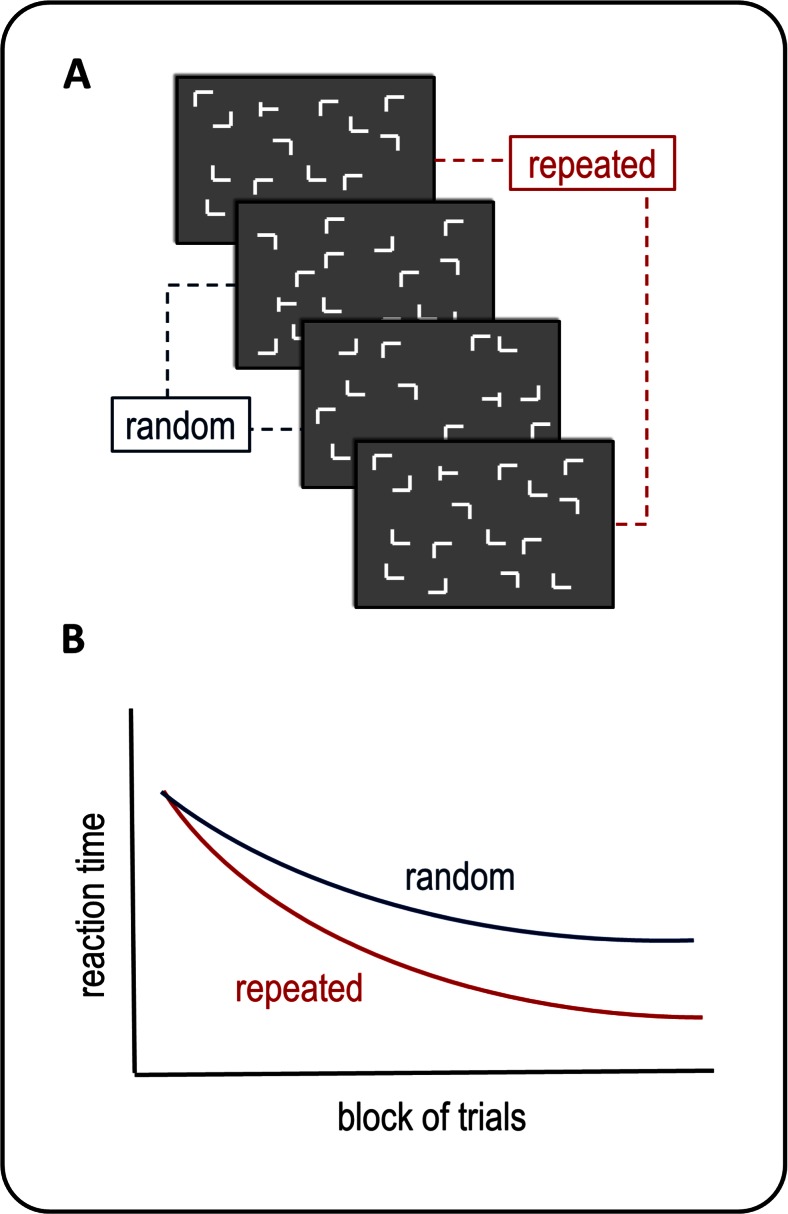
Panel A shows a sequence of search displays as used in standard contextual cuing experiments. Participants are instructed to search for a T-shaped target among a series of L-shaped distractors. Some search displays are regularly repeated during training, whilst others are new, unrepeated (random) displays. Panel B shows the typical pattern of results: Participants become faster at finding the target among the distractors in repeated displays

This problem is clearly illustrated by current research in a popular implicit learning paradigm known as contextual cuing (Chun & Jiang, 1998; Chun & Turk-Browne, 2008), which is the focus of the systematic review conducted here. In a typical contextual cuing experiment, participants are shown search displays containing a T-shaped target among a number of L-shaped distractors (see Fig. 1). The target is always rotated, so that the stem of the T points either to the left or to the right. Participants are instructed to find the T as fast as possible and report its orientation using two different keys. The search displays presented in half of the trials are repeated several times across training, while the remaining search displays are randomly generated in each trial, although participants are not informed about this manipulation. Across training blocks, participants’ reaction times (RTs) decrease systematically as they become familiar with the task. But, most importantly, this decrease is larger for repeated than for random search displays, indicating that across trials participants eventually learn something specific about the repeating patterns. That is to say, some mental representation is acquired of repeating displays which allows attention to be more and more rapidly deployed to the location where the target will be found (Chun & Jiang, 1998). This learning effect on RTs is highly robust and indeed is obtained in the vast majority of contextual cuing experiments.

Usually, the implicitness of this learning is assessed by means of a recognition test conducted at the end of the experiment. Participants are shown all the repeating patterns intermixed with new random patterns and are asked to report whether they have already seen each of those patterns. The learning effect found during the training phase is considered implicit if the number of patterns correctly recognized as old in the recognition test (hits) is no larger than the number of random patterns wrongly classified as old (false alarms), or if participants’ performance is at chance (50 % correct) overall. Another popular test used to assess whether learning was implicit is to ask participants to guess where the target would be in a search display where the target has been replaced by an additional distractor. If they perform at chance in this task, their learning about the repeating search configurations is again considered implicit. In both procedures, learning is assumed to be unconscious if a statistical comparison yields a null result.

However, as explained above, the statistical analyses typically conducted in these studies do not allow one to conclude that the null effects observed in the awareness tests reflect truly random performance. Meta-analysis across the whole body of experiments published in this domain permits us to check whether these null results reflect a real absence of awareness. Based on the relative proportions of significant results or on the overall trends of mean performance in awareness tests it is possible to measure to what extent the prevalence of null results reveals a genuine absence of awareness or merely insensitivity of statistical data in individual studies.

## Proportion and distribution of significant results

To assess to what extent the null results observed in these analyses reflect a real absence of awareness or a mere lack of statistical sensitivity, we conducted a systematic review of the literature. As explained in Appendix 1, we included in our analyses all the experiments that found spatial contextual cueing and that included either of the two awareness tests explained above (i.e., a recognition test or a target guessing test).

By definition, research on implicit processing assumes that participants lack awareness of the relevant regularity, and accordingly 78.5 % of the awareness tests yielded nonsignificant (*p* > .05) differences. However, 21.5 % of the awareness tests did yield a significant difference, well above (binomial *p* < .001) the theoretical 5 % of false positives that should be observed if the one-tailed null hypothesis is true with a standard α = .05. This proportion of significant results becomes particularly striking if we take into account that most of these statistical contrasts actually relied on two-tailed *t*-tests, for which the theoretical proportion of false positives would be just 2.5 %. The proportion of significant (*p* < .05) or marginally significant (.05 < *p* < .10) results was 27.6 %, again above the theoretical 10 % that would be predicted on the null hypothesis given a one-tailed test, binomial *p* < .001.

Regardless of the results of the inferential analyses, we also coded for each study whether participants performed numerically above chance (+1), exactly at chance (0), or below chance (-1) (see Appendix 1 for further details). The mean value of this direction score across experiments was 0.53 (95 % CI 0.41–0.66), far above the theoretical 0 that should be observed under the null hypothesis, *t*(165) = 8.468, *p* < .001, *d*_*z*_ = 0.66. The proportion of experiments scoring 1 was 66.9 %, significantly above 50 % in a binomial test, *p* < .001. Interestingly, within our database, the vast majority of experiments that reported a significant result had direction scores of 1. A logistic regression confirmed that there was a relationship between the direction scores and the probability of a significant result in the awareness tests, *B* = 1.37, *SE*_*B*_ = 0.483, Wald = 8.114, Odds ratio = 3.95, Model *χ*^2^(1) = 16.11, *p* < .001. In other words, significant results were far more likely to be associated with numerically above- than below-chance performance in the awareness test.

Overall, these results are not consistent with the idea that the null hypothesis reflects the true distribution of results in the awareness tests. On a true null hypothesis (hits = false alarms in the awareness test, or performance equal to chance), only around 5 % of studies should yield a significant result, and the number of effects in the “explicit” direction should equal those in the wrong direction. There should be no tendency for significant awareness results to be more prevalent in one direction than the other.

## Is there publication bias in the results of awareness tests?

However, it is still possible that the null hypothesis is true and that the unusually large number of significant results reflects a bias favoring the publication of significant results versus non-significant results. Even if participants perform at chance in the awareness test, occasionally the statistical analyses will yield a significant result by mere chance. If researchers or journals are biased towards publishing significant results, then the proportion of these in the published literature will exceed the theoretical proportion of false alarms that would be expected under the null hypothesis. Although this hypothesis might appear counterintuitive given that truly implicit learning requires null awareness, it is important to evaluate this possibility within the studies included in the meta-analysis.

Deviations from chance are more likely to occur in low quality experiments where the measurement error is larger (e.g., smaller samples or unreliable methods). That is to say, under the null hypothesis, large *and* significant effect sizes are more likely to be obtained in low- than in high-powered experiments. In meta-analyses, this trend is usually represented by means of a funnel plot representing the relationship between effect size and the measurement error. Unfortunately, it is difficult to draw a funnel plot with the information available in our dataset because many experiments did not report sufficient statistical information to compute effect sizes. For instance, standard errors and exact *t*-values were reported only in roughly half of the analyses. However, if publication bias were responsible for the unusually large number of significant results, then one would expect to find more significant results in low quality studies.

**Fig. 2:**
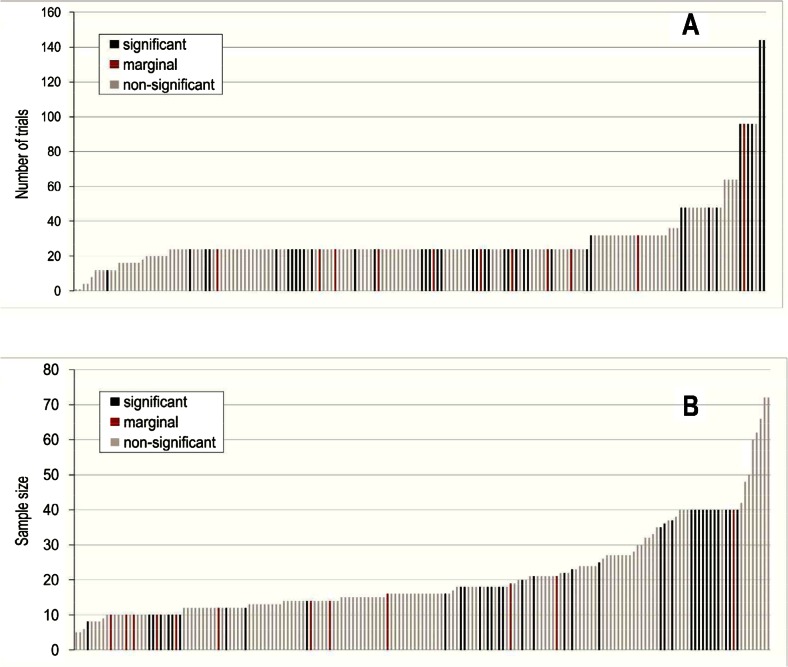
Contextual cuing experiments sorted by the number of trials of the awareness test (top panel) or by sample size (lower panel). Black bars denote statistical contrasts with significant results. Red bars denote statistical contrasts with marginally significant results

An important determinant of the quality of an experiment is the number of trials on which its measurement is based. The impact of random variance on the results can be minimized if a dependent variable is based on a larger number of observations. In the case of contextual cuing experiments, a large number of trials in the awareness test should yield less variable results and, therefore, a more precise measurement of awareness. Figure 2A shows the relationship between the number of trials and statistical significance. Dark bars represent significant (black) or marginally significant (dark red) results. The height of each bar represents the number of trials in the awareness test. As can be seen, if anything, the pattern of results is the opposite of what would be predicted on the basis of a publication bias: Null results are more prevalent among experiments including a small number of awareness trials. A logistic regression confirmed that the probability of finding a significant result increases as the number of trials increases, *B* = 0.024, *SE*_*B*_ = 0.009, Wald = 7.238, Odds ratio = 1.024, Model *χ*^2^(1) = 8.068, *p* = .005. Smyth and Shanks (2008) observed the same pattern in a single experiment: An awareness measure which was not significantly different from chance when based on 24 trials became significant when based on 96. The present results show that this pattern holds in aggregate across published studies.

Sample size, defined as the number of participants, is another important determinant of the methodological quality of an experiment. Studies conducted on larger samples are more likely to yield results that converge to the true effect size. Figure 2B shows the relationship between sample size and statistical significance in contextual cuing experiments. The height of each bar represents the sample size of the study. As in the case of the previous analysis, a logistic regression suggests that the probability of finding a significant result grows with sample size, *B* = 0.024, *SE*_*B*_ = 0.013, Wald = 3.247, Odds ratio = 1.024, Model *χ*^2^(1) = 3.128, *p* = .077. Although only marginally significant, this trend goes in the opposite direction from the one predicted if the high number of positive results were due to a publication bias favoring significant results over non-significant ones.

A defender of the implicit nature of contextual cuing could argue that awareness truly is absent in these studies, and that publication bias explains the prevalence of significant results in the meta-analysis. The results above show that this hypothesis is implausible and that the prevalence is not attributable to publication bias. However, they also show something else of importance, namely that many of the reported null results are likely to be false negatives arising from underpowered studies. As the quality of the measurement improves in terms of sample size and number of observations, it becomes appreciably more likely that the study will yield evidence of awareness.

## Effect sizes and statistical power

Overall, these analyses suggest that there is a true positive effect in the awareness tests employed in the studies included in the meta-analysis, and that failures to reach statistical significance are largely due to the small number of observations registered in most experiments, both in terms of sample size and in the number of trials included in the awareness test. Additional evidence for this interpretation can be obtained by exploring the typical size of the effect found in the awareness tests.

In many of the studies included in the present analyses, the authors failed to report sufficient information to compute the effect size of the results of the awareness test. Very frequently, the only piece of information available was that *p*-values were larger than .05, without additional details about *t*- or *F*-values. However, we were able to compute effect sizes for 96 of the statistical contrasts included in our data set. Based on sample sizes, reported *t*-values or, alternatively, one-degree-of-freedom *F*-statistics we were able to compute Cohen’s *d*_*z*_ effect size scores. We coded *d*_*z*_ scores as positive if the outcome went in the “explicit” direction (e.g., hit rate > false-alarm rate, regardless of significance) and as negative if the pattern of results was the opposite. Given the significant heterogeneity of effect sizes, *Q*(95) = 160.78, *p* < .001, we conducted a meta-analysis on *d*_*z*_ scores using a random effects model. The meta-analytic mean *d*_*z*_ was 0.31 (95 % CI 0.24–0.37).

Interestingly, although small, the meta-analytic effect size remains significantly greater than zero even if one actively removes from the meta-analysis all the statistical contrasts that turned out to be individually significant, *d*_*z*_ = 0.16 (95 % CI 0.10–0.22). Thus aggregate awareness is evident even amongst those studies that obtained no significant awareness and were on that basis interpreted as showing implicit learning. This speaks against the possibility that the studies in the meta-analysis represent two quite distinct sub-groups, one in which learning is truly conscious and one in which it is truly unconscious. Even when the true conscious studies are removed, the remainder yield above-chance awareness.

It is important to acknowledge the real size might be smaller than our meta-analytic estimate of *d*_*z*_ = 0.31. The *t*- and *F*-values were less likely to be reported when awareness tests failed to reach statistical significance, because in many of those cases the authors simply noted that *p*-values were larger than .05. Even so, assuming that 0.31 is approximately the true *d*_*z*_ of the typical awareness test, it is possible to compute what would be the required sample size to achieve a specific level of statistical power. Using G*Power 3 (Faul, Erdfelder, Lang, & Buchner, 2007) we found that, assuming a *d*_*z*_ of .31, a sample size of at least 66 participants would be needed to achieve statistical power of .80 in a one-tailed paired-samples *t*-test. For the more frequent two-tailed *t*-test, the figure goes up to 84. But recall that, as just mentioned, 0.31 might overestimate or underestimate the real effect size.

Most interestingly, the median *N* of all the contrasts included in the meta-analysis (also including the ones for which *d*_*z*_ could not be calculated) was 16. The statistical power of a sample of 16 participants to obtain a significant two-tailed effect given a *d*_*z*_ of 0.31 is around .21. Note that this range of statistical powers is virtually identical to the proportion of significant results (21.5 %) observed in our dataset. Given the small size of the effect found in the typical awareness test, the average sample sizes used in these studies are seriously underpowered. At the same time, the distribution of significant and nonsignificant results is close to what would be expected if the awareness results in individual studies are sampled from a distribution with a mean effect size of around .30.

## Effect size in implicit versus explicit measures

It might be countered that this effect size in the awareness test is far too small to account for the usually large contextual cueing effect found in these experiments, as the typical contextual cueing experiment yields effect sizes well above *d*_*z*_ = 1 on the implicit RT measure. If participants had conscious access to the representations learned in contextual cuing, why should this knowledge yield larger effects when assessed by means of visual search than when measured by means of an awareness test? This concern neglects the fact that contextual cuing and awareness are measured with radically different procedures. Even if they were measuring exactly the same memory trace, the differences between the procedures are so numerous that it would be naïve to expect the same effect size in both of them. Just to mention a clear difference, contextual cuing is traditionally assessed by gathering reaction times from hundreds of trials (usually more than 500 across the experiment). In contrast, awareness is assessed by means of just a few discrete responses. As can be seen in Fig. 2A, the number of trials rarely goes beyond 24 or 40. One cannot expect to find the same precision in a dependent variable based on a few observations of a discrete response as in one based on hundreds of observations of a continuous measure, even if those two dependent variables are measuring exactly the same latent variable.

In fact, when other constraints are taken into account, a small effect size is exactly what one would expect to find in any measure of contextual cuing that is not based on a very large number of observations. The available evidence shows that the faster reaction times found in repeated patterns are usually attributable to a small number of search displays (Schlagbauer, Müller, Zehetleitner, & Geyer, 2012; Smyth & Shanks, 2008). In other words, participants seem to learn very little or nothing about most of the search displays. Furthermore, it is also known that even for the search displays that elicit some learning, participants do not seem to acquire detailed information about all the elements in the search display. Instead, they seem to learn something only about the two or three distractors that happen to be closest to the target (Brady & Chun, 2007; Olson & Chun, 2002). Trying to detect these fragmentary memory traces in a brief recognition test, where each pattern is only presented once, is like finding a needle in a haystack. It is hardly surprising that the resulting effects are small.

**Fig. 3:**
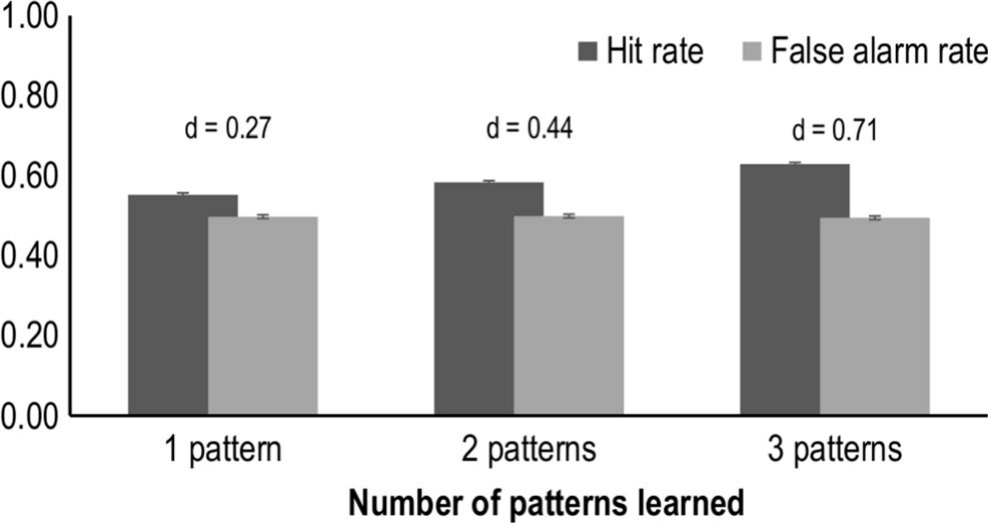
Results of a simulation exploring the size difference between hit rate and false alarm rate depending on the number of patterns learned by the participant. See the main text for more details. Error bars denote standard errors of the means across simulations

To further explore how small these effects can be, we conducted a simulation of the results which one could expect given these constraints. In a typical contextual cuing experiment, participants are exposed to 12 repeated patterns and 12 random patterns. In our simulation we assumed that participants would only be able to recognize one, two, or three of the 12 repeated patterns (for which they would therefore have a hit rate of 1.0) and that they would guess randomly when presented with any other pattern (either the 9–11 remaining repeated patterns or the 12 random patterns). Figure 3 shows the results of a simulation based on 1,000 simulated participants. As can be seen, the difference between the aggregate hit and false alarm rates is quite small in all cases. The tiny error bars shown in Fig. 3 refer to the standard error of the means across the 1,000 simulated participants. Using this small amount of sampling error as a yardstick, the Cohen’s *d* for the difference between hit rate and false alarm rate is only 0.44 for the case in which participants learn only two patterns. Even under the assumption that participants learn about three patterns it does not reach the conventional level for a large effect. It is not difficult to see that with just a small amount of additional measurement error, the effect size of these differences will be reduced to levels very similar to those found in our meta-analysis. That is to say, the small meta-analytic effect size is exactly what one would expect in a recognition test assuming that participants can only recognize correctly a couple of repeated patterns and that they guess whenever they are asked to identify a pattern that they do not recognize. The assumption that learning is based on only a small number of patterns is entirely consistent with what is known about the implicit learning effect in contextual cuing (Schlagbauer et al., 2012; Smyth & Shanks, 2008).

This simulation illustrates that the fact that the effect size of awareness is small does not mean that it is insufficient to explain or cannot be related to the (usually large) size of the contextual cuing effect found in reaction times. Instead, the small effect size found in awareness tests is exactly what one would expect to find when a subtle effect is assessed with an unreliable test. This problem does not apply to the usual measure of contextual cuing, which typically relies on hundreds of trials and consequently yields very precise estimations (and therefore large effect sizes) for even very subtle effects. The asymmetry between the small effects found in the awareness test and the large effects found in visual search facilitation can be attributed to differences in the sensitivity of the two measures (we return to this issue later).

It is interesting to note that the superior sensitivity of contextual cueing measures relative to awareness tests is also evident in experimental protocols where a brief awareness test is sufficiently powered to detect above-chance performance. For instance, it is widely acknowledged that contextual cueing is explicit when natural scenes are used as contexts. In these experiments (not included in our meta-analysis), a short test is usually enough to detect explicit awareness. But even so, this effect is disproportionally smaller than the corresponding contextual cuing effect found in reaction times. As an example, Brockmole and Henderson (2006, Experiment 1) found that participants performed above chance in a location-guessing test, and this effect was so large (*d*_z_ = 1.14) that it reached statistical significance with a small sample of only eight participants. But even this seemingly large effect is tiny compared to the huge size of the contextual cueing effect (*d*_z_ = 6.54). Thus, the reduced sensitivity of awareness tests is obvious even in experiments where learning is unambiguously considered explicit and tests are adequately powered to detect above-chance awareness.

## Confidence intervals as a partial solution to the false-negative problem

**Fig. 4:**
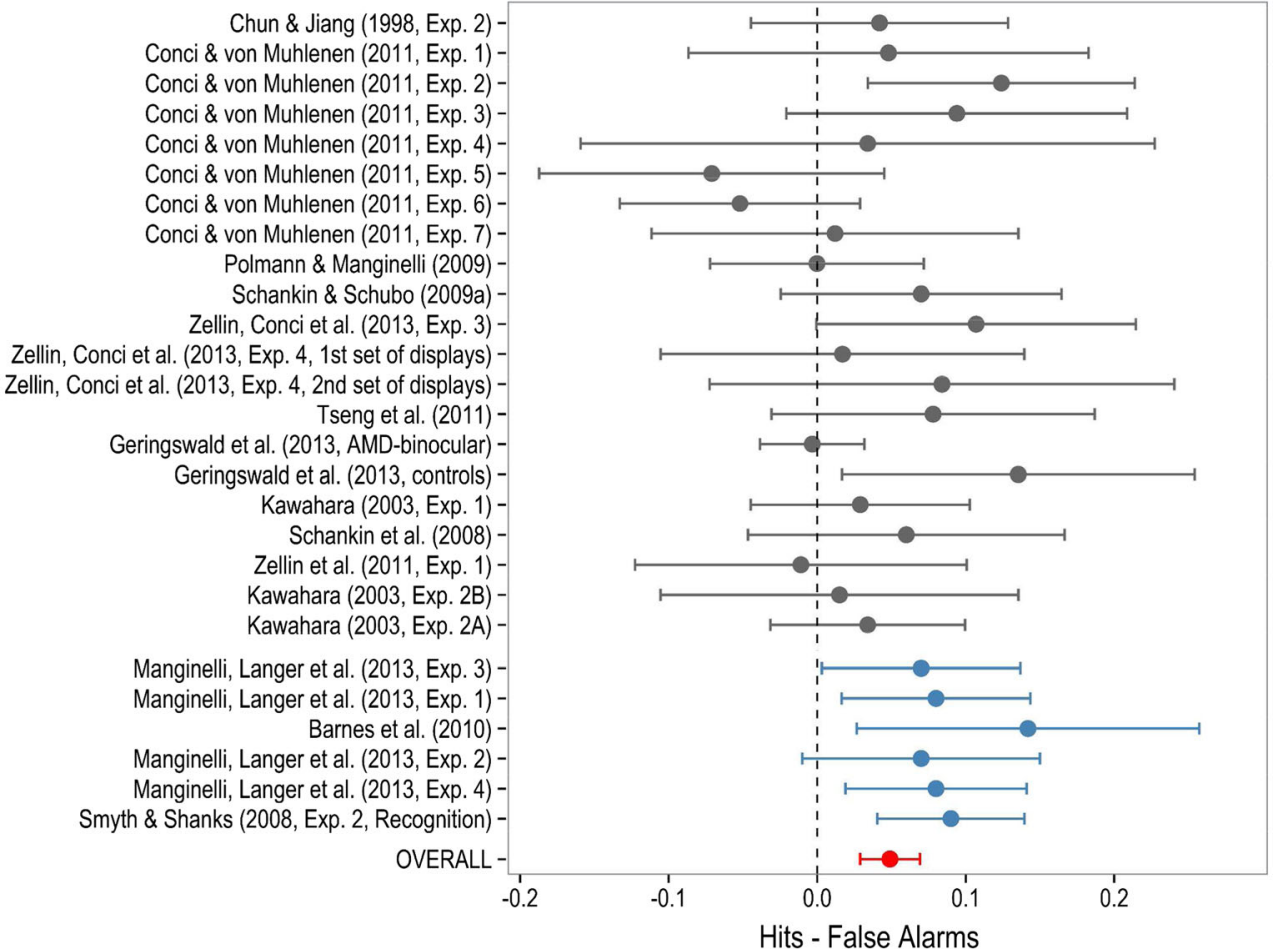
Ninety-five percent confidence intervals (CIs) of a subset of experiments contrasting hit rate versus false alarm rate in recognition tests. Given the heterogeneity of the studies included in the figure, *Q*(26) = 43.73, *p* = .016, the meta-analytic mean and CI shown in the last row were computed using a random effects model

It is easy to understand how null results can be false negatives by visually examining the CIs of the dependent variables. Figure 4 shows CIs for studies that employed a recognition test and that reported the mean hit and false alarm rates, and a *t*- or *F*-value. This figure does not aim to summarize the full results of the previous meta-analysis. It is offered only as a way of illustrating the misleading impression produced by null results. For the sake of simplicity we only show the CIs of studies with the typically small samples used in contextual cuing experiments (*N*s between 14 and 18) and experiments with relatively large samples (*N*s of 36 and above). All the experiments that meet these criteria are shown in Fig. 4.

Recall that a positive difference indicates that the proportion of hits was larger than the proportion of false alarms, in other words that participants were able to discriminate repeated from random search displays. As can be seen, for many of the studies with small sample sizes (19/21), the CI includes zero. Those results are usually taken as a proof that participants were unaware of learning. However, in general, these CIs are very wide. They include not just a small region around zero, but also a wide range of positive values. Therefore, these studies do not allow one to conclude that participants were unaware. They simply demonstrate that these experiments do not permit the level of awareness to be estimated with any precision.

In contrast, among the six experiments with the largest sample sizes the CIs are narrower and only one of them includes zero. Interestingly, the meta-analytic 95 % CI of all the experiments included in the figure overlaps with the CI of every single study. In other words, although the larger experiments yield significant results and the smaller experiments tend to yield non-significant results, there is actually no contradiction between them. Null results create the illusion that there is no difference between hits and false alarms and that participants were, therefore, unaware of learning. But the CIs do not allow this inference to be made with any degree of certainty. The use of CIs and graphic depiction is a powerful method for conveying the degree of precision in the estimate and of avoiding the temptation to interpret a failure to reject the null as evidence in favor of the null (Cumming, 2014; Fidler & Loftus, 2009).

## Bayes Factors as an alternative solution

CIs and meta-analysis provide a particularly clear and simple means to illustrate the uncertainty associated with underpowered studies, especially when the goal of the researchers is to draw conclusions on the basis of null results. However, an important shortcoming of CIs is that they fail to quantify the extent to which the results of an experiment favor the null or the alternative hypothesis. If an experiment yields a precise (i.e., narrow) CI around zero, it is legitimate to conclude that the null hypothesis is probably supported by the data, or at least that the effect is of little practical significance. But in the absence of a means to quantify support for the null hypothesis precisely this judgment remains somewhat arbitrary and subjective.

In contrast, Bayes Factors provide such a means to quantify the extent to which evidence favors the null or the alternative hypothesis and could accordingly play an important role in future research on contextual cuing and other implicit learning effects (Dienes, 2015). Specifically, a Bayes Factor (*BF*_*10*_) represents the ratio between the likelihood of the data given the alternative hypothesis (1) and the likelihood of the data given the null hypothesis (0). A *BF*_*10*_ larger than 3 is usually considered to reflect substantial support in favor of the alternative hypothesis and values larger than 10 strong support. Conversely, values lower than 1/3 are considered substantial evidence and values lower than 1/10 strong support for the null hypothesis (Wetzels, Matzke, Lee, Rouder, Iverson, & Wagenmakers, 2011).

Do the results of the awareness tests reviewed in our meta-analysis provide more support for the null hypothesis than for the alternative hypothesis? To answer this question, we converted all the 96 effect sizes entered in the meta-analysis back to *t*-values that we submitted to a Bayes Factor analysis using a Cauchy distribution with a (default) scaling factor *r* = 0.707 as the alternative hypothesis. To improve the comparability of values supporting the null hypothesis (originally bounded from 0 to 1) with values supporting the alternative hypothesis (originally bounded from 1 to ∞), we took the logarithm of all *BF*_*10*_’s, which yields a symmetric distribution where all negative values support the null hypothesis and all positive values support the alternative hypothesis. On this logarithmic scale, values roughly larger than 1.1 provide substantial support for the alternative hypothesis (*BF*_*10*_ > 3) and values roughly larger than 2.3 provide strong support (*BF*_*10*_ > 10). Conversely, values lower than −1.1 or than −2.3 constitute substantial and strong support for the null hypothesis.

**Fig. 5:**
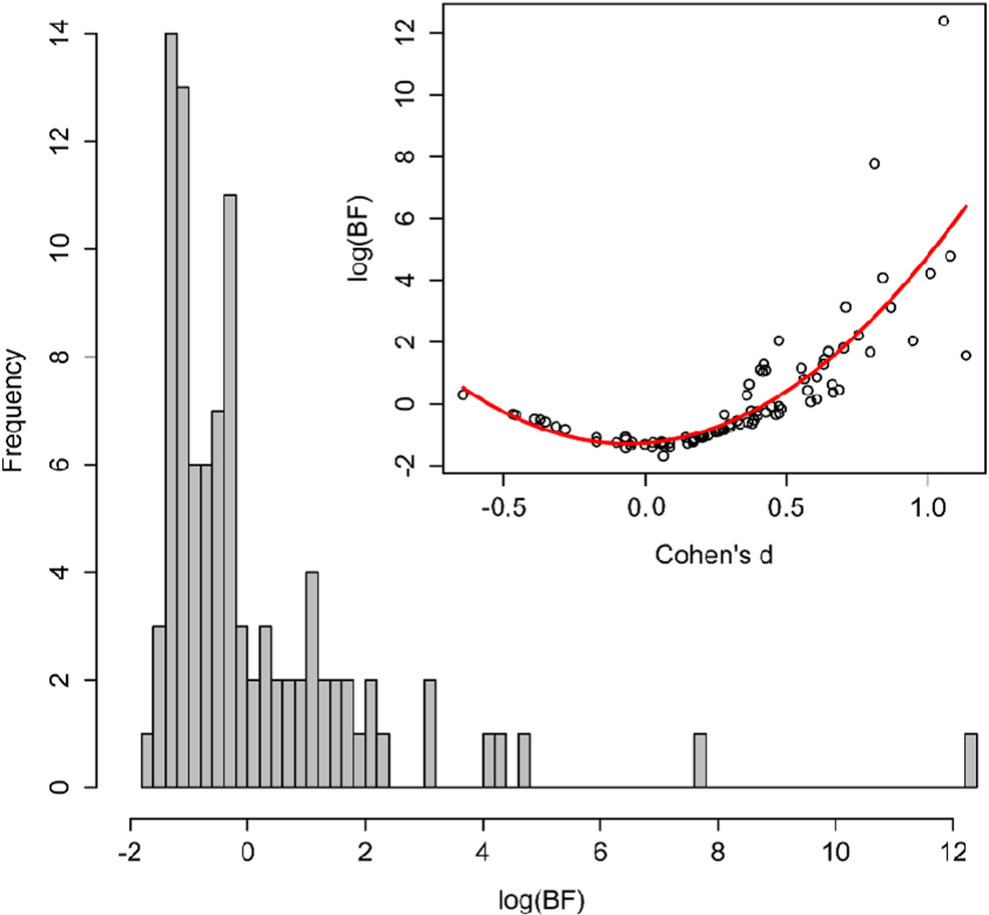
Histogram of the logarithmic Bayes Factors (*BF*_*10*_’s) included in the meta-analysis. Positive values indicate support for the alternative hypothesis (awareness) and negative values indicate support for the null hypothesis (unawareness). The inset depicts a scatterplot of effect sizes (Cohen’s *d*) and logarithmic *BF*_*10*_’s with the best fitting quadratic function

The resulting distribution of the log(*BF*_*10*_)’s is depicted in Fig. 5. Interestingly, this distribution offers some encouragement for the view that contextual cueing can be implicit. The majority of results provide some support for the null hypothesis over the alternative hypothesis, suggesting that learning was indeed unconscious in many of these studies. However, a closer inspection of Fig. 5 also reveals an important asymmetry between the positive and negative values. While positive values span a wide range of values (providing not just substantial but even strong evidence for the alternative hypothesis), negative values rarely go beyond −1 or −1.50 and they never reach the −2.30 boundary. In other words, many studies yield *BF*_*10*_’s more consistent with the null hypothesis (no awareness), but the weight of this evidence is never strong. For the sake of clarity, Fig. 5 also includes a scatter plot depicting the relationship between *BF*_*10*_’s and effect sizes, together with the best fitting quadratic function. Consistent with the assessment above, the vertex of this quadratic function, which seems to capture well the typical lower values of log(*BF*_*10*_), is equal to −1.28, corresponding to an unconverted *BF*_*10*_ = 0.27.

Therefore, this Bayesian analysis offers a somewhat tantalizing view of the implicitness of contextual cueing that has important implications for future research: On the one hand, there are a large number of studies with results numerically more consistent with the null hypothesis (no awareness) than with the alternative hypothesis (awareness). On the other hand, there are more experiments strongly supporting the alternative hypothesis than strongly supporting the null hypothesis. Fortunately, Bayesian statistics also offer a way of resolving this apparent contradiction regarding the inconclusiveness of existing evidence. Although in NHST researchers are not free to continue testing participants after reaching the sample size they specified a priori, Bayesian statistics do allow researchers to continue gathering data (e.g., in an awareness test) until a specific level of precision is reached (Dienes, 2011, 2014), for instance, until the Bayes Factor becomes larger than 10 or smaller than 1/10. This feature of Bayesian statistics make Bayes Factors a powerful means by which future research could establish the implicitness of contextual cuing and other seemingly unconscious learning effects (Rouder, Morey, Speckman, & Pratte, 2007).

## Correlations and post hoc data selection

We should acknowledge that many of the studies included in the meta-analysis based their conclusion – that the contextual cuing they obtained was implicit – not only on a null result in an awareness test but also on one of two additional pieces of evidence (or both): Correlations and *post hoc* data selection. However both of these are statistically problematic.

The first of these refers to the finding that across participants, the magnitude of contextual cuing tends not to be significantly correlated with the measure of awareness. For instance, going back to the examples depicted in Fig. 4, Zellin, Conci, von Mühlenen, and Müller (2013, Experiment 3) found a marginally significant effect in the awareness test. However, instead of concluding that learning was explicit, they went on to estimate the correlation between the results of the awareness test and the size of contextual cueing and found a correlation of *r* = .42, *p* > .10. This lack of significant correlation seems on the face of it to provide further and stronger support for the claim that learning is implicit, but a moment’s thought reveals that once again absence of evidence is not the same as evidence of absence. Without knowing the CI on the correlation coefficient, we cannot evaluate how much weight to place on the null result, yet authors never report such CIs. We computed the 95 % CI on the correlation coefficient obtained by Zellin, Conci et al. (2013, Experiment 3) and found that it had lower and upper limits of −.14 and .77. Thus the data in this study are compatible with a true correlation as large as .77 or as low as −.14. Similarly, Conci and von Mühlenen (2011, Experiment 2) and Preston and Gabrieli (2008) reported non-significant correlations with 95 % CIs of [−.42 to .62] and [−.33 to .49], respectively. Obviously, these estimations are too imprecise to permit any strong conclusions to be drawn.

Furthermore, it is common practice to report the correlation between explicit and implicit measures of learning only when the explicit awareness measures yield significant results (e.g., Conci & von Mühlenen, 2011, Experiment 2; Geyer, Shi, & Müller, 2010; Peterson & Kramer, 2001; Preston & Gabrielli, 2008). This is particularly problematic. In just the same way that multiple testing increases the risk of type 1 errors, it also increases the risk of type 2 errors. Put differently, if researchers explore different awareness measures until they find one that yields a null result, the chances that the null result will reflect a false negative increase as the number of statistical tests grows. To prevent type 1 errors when multiple comparisons are conducted it is usual to make adjustments of *α*, like the Bonferroni correction. Similarly, in order to prevent type 2 errors, it would be necessary to adjust *β* for multiple comparisons, which is virtually identical to increasing statistical power, defined as 1- *β*.

We have argued here that studies which measure awareness alongside some “implicit” behavioral measure can yield erroneous evidence if NHST leads researchers to mistake weak awareness for null awareness. We have also noted that this problem applies not only to the interpretation of the awareness measure itself and whether it exceeds chance, but also extends to interpretation of correlations between implicit and explicit measures where absence of evidence can again be misinterpreted as evidence of absence. One final method may at first sight appear to avoid these problems by unequivocally ensuring null awareness: Selecting participants *post hoc* who score at or below chance on the awareness measure. If such a sample of participants (or a sample of configurations) shows significant contextual cuing (which they do: e.g., Colagiuri, Livesey, & Harris, 2011; Geyer, Shi, & Müller, 2010; Geyer, Zehetleitner, & Müller, 2010; Smyth & Shanks, 2008), then surely this is clear evidence of true implicit learning? The answer to this question is an emphatic “no.” The method is statistically unsound (Shanks & Berry, 2012).

To see this, we demonstrate that the pattern can arise even when the awareness and behavioral measures are based on the very same underlying representation or latent variable. We assume that a contextual cuing experiment gives rise to a participant acquiring knowledge of the repeating (compared to novel) configurations that we can capture by the memory strength variable *s*, which is normally distributed with mean and standard deviation (*SD*) equal to 1, and with *s* = 0 representing the baseline of no configuration knowledge. This common underlying knowledge forms the basis of *both* the “implicit” behavioral RT measure *and* the recognition awareness score, measured as effect size *d* computed from recognition hits minus false alarms. Specifically:

1 $$ \mathrm{R}\mathrm{T}=100s+30e $$

2 $$ d=0.30s+e $$

**Fig. 6:**
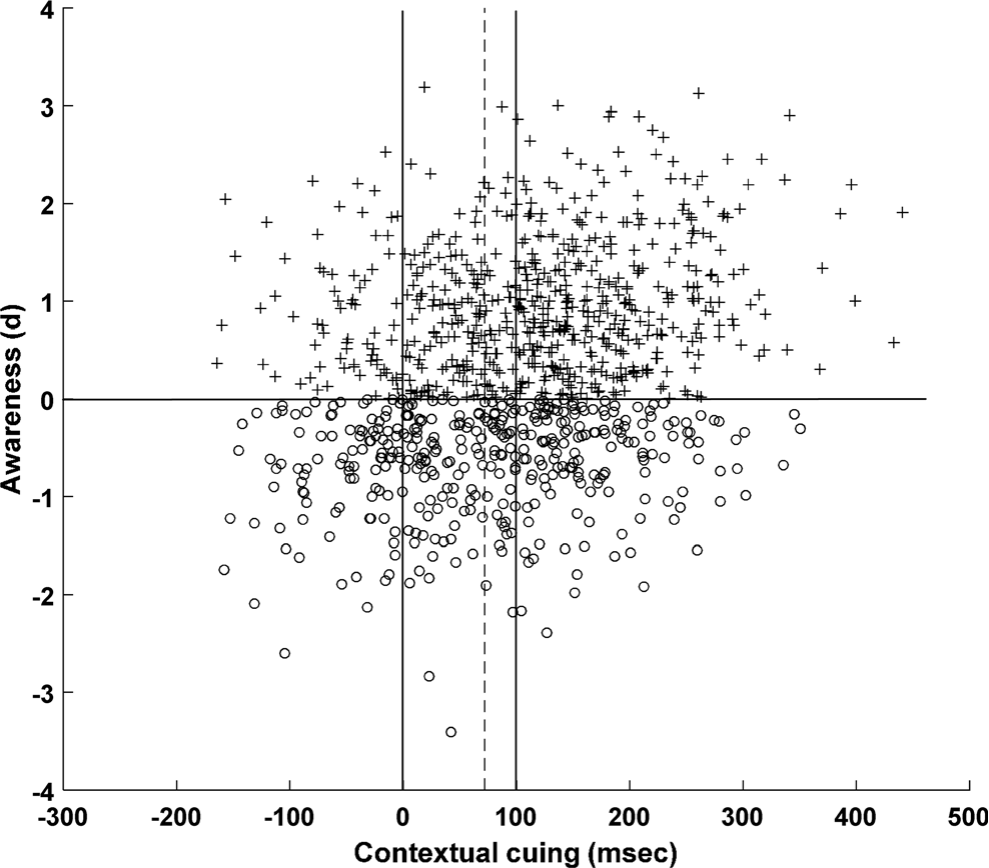
Contextual cuing (msec) plotted against awareness (recognition hits minus false alarms, expressed in terms of effect size *d*) in 1,000 simulated participants. Mean contextual cuing across the entire sample is 100 msec (rightmost vertical line), while that in the subset of simulated participants scoring at or below *d* = 0 (open circles) is approximately 70 msec (dotted vertical line)

A given participant is assumed to have knowledge of the repeating patterns, *s*, which is first scaled by a factor of 100 in Eq. 1 and combined with some normally distributed random error *e* which has a mean of zero and *SD* of 1 to yield that participant’s implicit contextual cuing RT effect. This very same value of *s* is scaled by a factor of 0.3 in Eq. 2 and combined with independent error (it is important to emphasize that while the same value of *s* features in the two equations, the noise *e* added in each case is independent) to yield that participant’s explicit awareness score. Figure 6 shows data generated by this simple model for 1,000 simulated participants. Because of the chosen scaling factors, participants generate a mean contextual cuing RT score of 100 msec, which is roughly the level seen in contextual cuing experiments, and a mean awareness score of 0.30, consistent with the meta-analytic effect. The two measures are weakly correlated, *r* ≈ 0.3, again consistent with the data.

We now select only those simulated participants who individually score at or below chance (*d* = 0) on the awareness measure (illustrated by the open circles in Fig. 6) and we ask what contextual cuing score we see in these “unaware” participants. The score in these participants is ~70 msec. Despite the fact that contextual cuing and awareness are based on the same underlying knowledge representation in this model (and on nothing else apart from noise), and that these participants are selected on the basis of chance (or below chance) awareness, they nonetheless show a highly reliable contextual cuing effect. There is no mystery to this: It is simply a manifestation of regression to the mean. In noisy bivariate data, a sample created by applying a cut-off on one dimension will have a mean on the other dimension that is closer to the overall mean. Note that although this demonstration concerns participants selected *post hoc*, the same logic applies to configurations selected in the same way (e.g., Geyer, Shi, & Müller, 2010; for a similar approach, see Conci & von Muhlenenn, 2011, Experiment 2). It implies that the logic of interpreting significant contextual cuing in participants (or configurations) retrospectively chosen because their awareness is at or below chance as evidence of implicit learning can be misleading.

Lastly, note that across all of the data generated by the model, the effect size for contextual cuing is Cohen’s *d* ≈ 1 while that for awareness is *d* ≈ 0.3 (these can be calculated directly from Eqs. 1 and 2). Thus, confirming what we claimed earlier, the fact that real studies might yield larger effect sizes for contextual cuing than for awareness does not license the conclusion that the former is based on some special form of unconscious knowledge. It arises simply because the model assumes a greater relative contribution of random error to awareness measures than to contextual cuing.

## Conclusions drawn by authors and impact on publication quality

The analyses conducted so far give us reasons to suspect that many, if not most, of the null results obtained in this kind of awareness test can be considered false negatives. This conclusion stands in stark contrast with the certainty with which authors interpret these null results as strong evidence in support for the null hypothesis. As an example, the experiment with the widest CI in Fig. 4 is Experiment 4 from Conci and von Mühlenen (2011). In spite of the uncertainty revealed by the CI of the awareness test, the conclusion drawn by the authors was that “no explicit awareness of the display repetitions could be formed” (Conci & von Mühlenen, 2011, p. 219). Note also the results of the two conditions analysed in Experiment 4 of Zellin, Conci et al. (2013). Although they include zero, the CIs do not exclude a wide range of positive values. Obviously, no conclusion can be drawn with any assurance from the results of those awareness tests. However, the interpretation of the authors was that “observers did not explicitly recognize the old context-displays” (Zellin, Conci et al., 2013, p. 10).

**Fig. 7:**
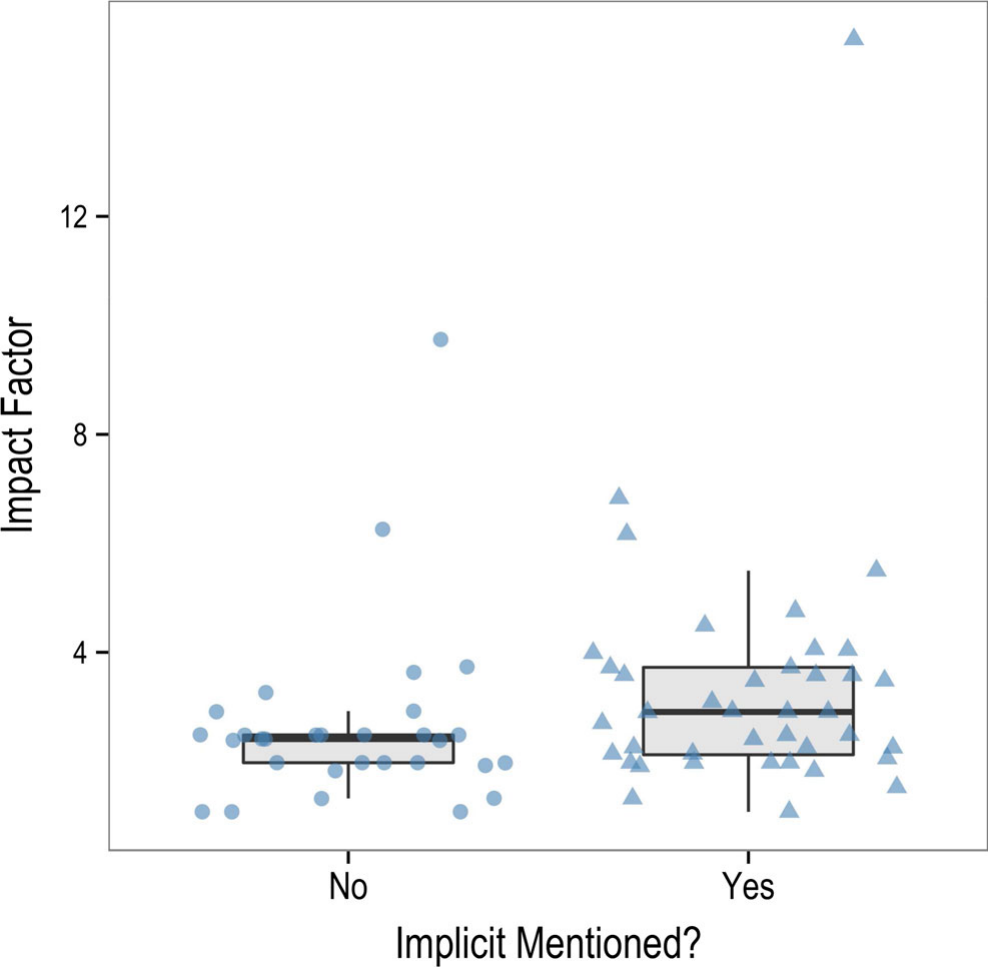
Impact factor of journals that published papers mentioning or not mentioning “implicitness” in the title

Researchers can hardly be blamed for their tendency to over-interpret these null results as reflecting a genuine absence of awareness. The “implicit” status of contextual cuing is probably one of the features that make it most attractive and salient to the scientific community. As can be seen in the list of studies included in the meta-analysis, the titles of most articles include some allusion to the implicit or automatic nature of contextual cuing. In fact, 42 of the 73 articles included in this analysis mention the concept of implicitness in their titles. There are obvious reasons for the emphasis on the implicit character of contextual cuing. Figure 7 depicts the impact factors of the journals in which the articles analysed here were published, depending on whether they mentioned implicitness (“implicit,” “explicit,” “awareness,” “unconscious,” or “recognition”) or not in the title. Three of the 73 articles could not be included in Fig. 7 because they were published in journals of books not included in *Journal Citation Reports*. Given that the distribution of impact factors was highly skewed, we logarithmically transformed them. As suggested by Fig. 7, the difference in mean impact factor between articles mentioning and not mentioning implicitness was statistically significant, *t*(68) = 1.98, one-tailed *p* = .026, *d* = 0.48, suggesting that papers mentioning implicitness in their titles made their way into higher impact-factor journals. Although this result is no more than correlational at best, it does provide some hint about the incentives that exist for interpreting contextual cuing as unconscious.

## Conclusions

In recent years the scientific community has witnessed growing concern about the high rate of false positives and unreliable results within published studies (Francis, 2012; Ioannidis et al., 2014; Simonsohn, Nelson, & Simmons, 2014). In contrast, the potential impact of false negatives has remained largely ignored (Fiedler et al., 2012). This asymmetry is natural, given that most experiments seek to observe positive results. However, there are many areas of psychological research where the evidential value given to null results is critical. In fact, there are several reasons to suspect that the over-interpretation of null results is even more dangerous than the prevalence of false positives in some areas of research. First, null results are inherently ambiguous. They indicate that there is not enough support for the alternative hypothesis, but they are silent about the amount of support for the null hypothesis. Second, unlike positive results, null results are surprisingly easy to obtain by mere statistical artefacts. Simply using a small sample or a noisy measure can suffice to produce a false negative.

The results of the present systematic review suggest that these problems might be obscuring our view of implicit learning and memory in particular and, perhaps, implicit processing in general. It is popularly claimed that contextual cuing and other implicit learning effects take place without participants becoming aware of the representations they learn (Chun & Jiang, 2003). Contrary to this prevalent view, we found that the seemingly chance-level performance of participants in awareness tests is more likely to reflect a type 2 error. The overall proportion of positive results is too large for the null hypothesis to be true. This proportion cannot easily be explained in terms of publication bias favoring positive results, but is perfectly consistent with the frequency of positive results that one would expect to find, given a true but modest-sized awareness effect, in underpowered experiments using unreliable dependent measures. This result is also consistent with experimental evidence suggesting that the quality of the awareness test is a key determinant of whether contextual cuing experiments yield “explicit” or “implicit” results (Smyth & Shanks, 2008).

We have offered some suggestions about how future studies could provide firmer evidence for implicit learning in contextual cuing, including increasing sample sizes to boost power, reporting CIs, and continuing to collect awareness (e.g., recognition) data until the Bayes Factor crosses a boundary of evidential support. We have also suggested that two data analytic techniques should unequivocally be avoided in future studies: The calculation of implicit-explicit correlations after finding that the implicit score is significantly greater than chance, and *post hoc* data selection.

Before ending, we would also like to emphasize that we do not believe that researchers working in this area are following these practices (e.g., using small numbers of testing trials or relying on NHST to claim support for the null hypothesis) in a deliberate attempt to deceive their readers. Most likely, researchers are simply following routinely a research protocol that, with its pros and cons, has become standard. It must be acknowledged that many of the experiments included in the present meta-analysis (and especially those that made no mention of awareness in their titles) were designed primarily to explore issues largely unrelated to the question of whether contextual cuing is implicit or not, such as the role of working memory in contextual cueing, how spatial associations are formed, the neural underpinnings of contextual learning, and so on. The fact that awareness was only a secondary concern might explain why the vast majority of them did not include a sensitive (and lengthy) awareness test and why they relied on simple NHST to analyse their results. But this only serves to illustrate how easily a particular conception can gain momentum in a substantial body of literature and become part of the *zeitgeist*, despite weak evidence.

Although we restricted our analyses to experiments conducted within a specific implicit learning paradigm, the same problem extends to other phenomena where participants’ awareness is discounted on the basis of NHST, such as subliminal perception and other forms of unconscious learning and implicit processing that we have not considered here (e.g., Dehaene et al., 1998; Pessiglione et al., 2008). False negatives also pose important problems for current attempts to replicate controversial findings.

These and other examples show that null results in underpowered studies may give the false impression that an effect is genuinely absent when actually it is not. They can also create the impression that there is a deep inconsistency between studies showing significant results and those yielding null results, even when the latter just reflect a lack of statistical sensitivity. Fortunately, researchers can resort to alternative statistical analyses when they need to assess the amount of support for the null hypothesis, including CIs, Bayes factors, and counternull values (Cumming, 2014; Dienes, 2015; Rosenthal & Rubin, 1994; Rouder et al., 2009). The price we pay for our reluctance to use these alternatives to NHST is that important aspects of what we believe about cognition may be mistaken.

## Appendix 1

### Literature search strategy

For the present systematic review, we accessed all the published reports that used the standard procedure for contextual cuing experiments. On 26 November 2013 we searched in the Web of Science for all the papers citing the original report from Chun and Jiang (1998). Based on the contents of the abstracts available on the Web of Science, we removed from this list theoretical reviews with no empirical work and also empirical papers focused on topics different from contextual cuing. We also removed contextual cuing papers that used natural scenes as contexts because the cognitive processes involved in these experiments are widely recognized to be explicit (Brockmole & Henderson, 2006; Brockmole & Vo, 2010).

Within the remaining list of studies, we were particularly interested in experiments whose general procedure did not deviate radically from the standard method described in the Introduction. Specifically, we selected all the experiments in which the location of the distractors in repeated displays predicted the location of the target within the same static display. This criterion excluded a small set of experiments on identity cuing, temporal cuing, contextual cuing with moving patterns, and also experiments in which distractors predicted the location of a target presented on a subsequent search display. Finally, by studying the reference lists of the accessed reports, we identified a small group of relevant papers that had not appeared in our original search in the Web of Science (Geringswald, Herbik, Hoffman, & Pollmann, 2013; Manns & Squire, 2001; Pollmann & Manginelly, 2010; Zellin, von Mühlenen, Müller, & Conci, 2013) and we included them among the final list of studies. Following this procedure we identified 73 articles that contained at least one experiment that qualified for the present meta-analysis. All the papers included in our review are marked with asterisks in the References section.

### Selection of experiments, conditions, and statistical tests

Only experiments including an awareness test (either a recognition test or a target guessing test) were considered in the present analysis. We ignored data from experiments in which contextual cuing was not observed. Similarly, if an experiment comprised several conditions and awareness test results were reported separately for each condition, we only analysed awareness tests from those conditions that yielded contextual cuing.

If the authors conducted several awareness tests (e.g., a recognition test and a target-location guessing task) we included all of them in our analyses. Because we were interested in the role of the number of trials included in the awareness test (see main text), we included several analyses of the same condition only when these were based on blocks of trials of different sizes. For instance, if one experiment included an awareness test with two blocks of 24 trials each and the analyses were conducted on block 1, block 2, and blocks 1 and 2 collapsed, we included all three contrasts in our analyses, coding the number of trials of each of them as 24, 24, and 48 respectively. In contrast, if the authors reported multiple analyses of the results of a single awareness test, we only included one of them, for instance, if the same data set was first used to compare hit rate versus false alarm rate and then to compare overall performance against chance. In cases like these, comparisons of hits versus false alarms were favored over alternative analyses of the same data (such as *d’* scores or comparisons of performance against chance). The logic behind this selection strategy is to extract several analyses from a single experiment when they conveyed independent information (different number of trials or different awareness tests) but not when they were mere re-analyses of exactly the same data set. Following these criteria, we obtained data from 181 statistical contrasts.

### Coding of study characteristics and results

We were particularly interested in knowing whether the results of the awareness tests were statistically significant or not. Therefore, we coded non-significant results (*p* > .05) as 0 and significant results (*p* ≤ .05) as 1. If the reported data allowed the reader to infer that a result was marginally significant (.05 < *p* < .10) we coded that result as 0.50. Regardless of the significance of the statistical contrast, we also coded whether the descriptive pattern of results went in the “explicit” direction (e.g., hit rate > false alarm rate) or in the opposite direction. We coded studies in the “explicit” direction as 1, studies in the opposite direction as −1, and studies in which hits and false alarms were equal (or in which participants performed exactly at chance level) as 0.

For each contrast we were interested in knowing the number of participants on which the contrast was based and the number of trials included in the awareness test, as these determine the power of the experiment to detect non-zero awareness. These two variables were also coded in our datasets. The number of participants of one experiment (Colagiuri et al., 2011) was an outlier (*z* > 12) and was recoded as the number of participants of the next largest experiment included in the data set. Similarly, the number of trials of the awareness test conducted in another experiment (Geyer, Shi, & Müller, 2010, Experiment 3) was an outlier (*z* = 11.97) and was recoded as the number of trials of the experiment with the next largest number of trials in the data set. This recoding strategy is common practise in meta-analytic reviews (e.g., Hofmann, Gawronski, Gschwendner, Le, & Schmitt, 2005). The qualitative conclusions of our analyses are not altered by using the actual number of participants.

Finally, although not all experiments included sufficient information to compute an effect size estimate, when these data were available, we did collect them. In the case of 96/181 (53 %) contrasts, we were able to compute Cohen’s *d*_*z*_ scores from *t*-values or *F*-values with one degree of freedom. We computed *d*_*z*_ scores by dividing *t*-values by the square root of the relevant sample size (note that the contrast between hits and false alarms or between performance and chance is within-participants for all studies in the meta-analysis). When only *F*-values with one degree of freedom were reported, we converted them to *t*-values. In a few cases, we detected a contradiction between the sample size reported in the paper and the degrees of freedom of the *t* and *F* contrasts. When this occurred, we computed the effect size taking the degrees of freedom reported in the statistical test as the correct estimate of the sample size. We ignored *t*-values from two experiments (Geyer, Shi, & Müller, 2010, Experiment 3; Geyer, Zehetleitner, et al., 2010, Experiment 1) because their degrees of freedom were reported as “*t*(1, 11),” making it unclear whether they used a *t* distribution with 11 degrees of freedom or an *F* distribution with 1 and 11 degrees of freedom. We also ignored four *t*-values where the reported data did not allow us to conclude whether the effect size was positive or negative. The random effects meta-analysis was conducted with the “metaphor” R package (Viechtbauer, 2010). Bayes Factors were computed with the “BayesFactor” R package (Rouder et al., 2009).

## Coding of paper and journal characteristics

As a proxy to measure the relevance of the implicitness of contextual cuing for each study, we coded whether the title of the paper made allusion to the implicit character of this effect. Papers were sorted depending on whether or not they mentioned the words “implicit,” “explicit,” “awareness,” “unconscious,” or “recognition.” We also coded the 2012 impact factor of the journals that had published the studies included in the present meta-analysis. Because of a change in the name of the journal, impact factors for *Attention, Perception, & Psychophysics* were also used for papers published in the journal with the previous name, *Perception & Psychophysics*. All impact factors were obtained from the 2012 edition of the *Journal Citation Reports*.
